# *Acinetobacter baumannii* among Patients Receiving Glucocorticoid Aerosol Therapy during Invasive Mechanical Ventilation, China

**DOI:** 10.3201/eid2812.220347

**Published:** 2022-12

**Authors:** Wenchao Zhang, Mei Yin, Wei Li, Nana Xu, Haining Lu, Weidong Qin, Hui Han, Chen Li, Dawei Wu, Hao Wang

**Affiliations:** Qilu Hospital of Shandong University, Jinan, China (W. Zhang, M. Yin, W. Li, N. Xu, W. Qin, H. Han, C. Li, H. Wang);; Qingdao Branch, Qilu Hospital of Shandong University, Qingdao, China (H. Lu, D. Wu)

**Keywords:** *Acinetobacter baumannii*, bacteria, antimicrobial resistance, aerosol inhalation, glucocorticoid, invasive mechanical ventilation, healthcare-associated infections, China

## Abstract

*Acinetobacter baumannii* is a nosocomial pathogen associated with severe illness and death. Glucocorticoid aerosol is a common inhalation therapy in patients receiving invasive mechanical ventilation. We conducted a prospective cohort study to analyze the association between glucocorticoid aerosol therapy and *A. baumannii* isolation from ventilator patients in China. Of 497 enrolled patients, 262 (52.7%) received glucocorticoid aerosol, and *A. baumannii* was isolated from 159 (32.0%). Glucocorticoid aerosol therapy was an independent risk factor for *A. baumannii* isolation (hazard ratio 1.5, 95% CI 1.02–2.28; p = 0.038). Patients receiving glucocorticoid aerosol had a higher cumulative hazard for *A. baumannii* isolation and analysis showed that glucocorticoid aerosol therapy increased *A. baumannii* isolation in most subpopulations. Glucocorticoid aerosol was not a direct risk factor for 30-day mortality, but *A. baumannii* isolation was independently associated with 30-day mortality in ventilator patients. Physicians should consider potential *A. baumannii* infection when prescribing glucocorticoid aerosol therapy.

## Abstract 2

Aerosolized glucocorticoid treatment was independently associated with bacterial isolation in these patients.

*Acinetobacter baumannii*, a gram-negative coccobacillus, is a major nosocomial pathogen worldwide. *A. baumannii* is particularly challenging in intensive care units (ICUs). According to the Extended Prevalence of Infection in Intensive Care study, aimed at providing information on the prevalence of infection in ICUs worldwide, *Acinetobacter* spp. constituted 8.8% of all culture-positive ICU infections in 2007 (*1*), which increased to 11.4% in 2017 (*2*). However, infection rates differed markedly, ranging from 1.0% in North America to 25.6% in Asia and the Middle East and 22.9% in eastern Europe (*2*). Patients on invasive mechanical ventilation are particularly vulnerable to *A. baumannii* infection and colonization due to airway barrier destruction and bacterial virulence factors such as motility, epithelial adherence, and biofilm formation that enable *A. baumannii* colonization in the airways (*3*,*4*). *A. baumannii* in patient airways is associated with longer hospitalization, higher medical expenses, and increased mortality rates (*5*–*7*). Identifying risk factors for *A. baumannii* infection is crucial for implementing preventive measures and decreasing overall illness and death.

Aerosol inhalation is widely used in patients requiring mechanical ventilation. Glucocorticoids are frequently administered during aerosol therapy, especially in China (*8*–*10*). Compared with systemic application, aerosol therapy has several advantages, including targeted delivery to the lungs, faster response, and fewer systemic side effects (*11*,*12*). However, the aerosols and droplets generated during aerosol inhalation can become sources of respiratory pathogens (*13*), and inhaled glucocorticoids might suppress pulmonary immunity (*14*), which could increase the opportunity for nosocomial acquisition. Inhaled corticosteroids are associated with an increased risk for pneumonia in patients with chronic obstructive pulmonary disease (COPD) (*15*). However, the effects of glucocorticoid aerosol inhalation on nosocomial infection risk has not been clearly elucidated.

Glucocorticoid aerosol therapy is mainly indicated for patients with asthma, COPD (*16*), acute respiratory distress syndrome (ARDS) (*17*), and some pathophysiological conditions, such as airway hyperresponsiveness (*18*), hyperinflammation, and mucosal edema (*19*). In the past decade, use of glucocorticoid aerosol therapy has increased in hospitals in China; on average, >40% of patients on mechanical ventilation receive this therapy (*9*). In addition, a market analysis determined that aerosolized glucocorticoid sales in China were almost 3-fold higher in 2018 than in 2012 (*20*).

Although epidemiology has demonstrated a slow increase in *A. baumannii* infection globally over the past decade (*1*,*2*), the increase in *A. baumannii* incidence in China appears to have outpaced increases in other regions worldwide (*21*–*23*). According to the China Antimicrobial Surveillance Network (CHINET), a national surveillance of the trends of bacterial strains isolated from the major hospitals in China, the number of *Acinetobacter* spp. strains increased by 2.7-fold in 2018 compared with 2012 (*23*,*24*). Previously, we reported a marked increase in the incidence of *A. baumannii–*related bloodstream infections and incidence of pneumonia-related *A. baumannii *infections in ICUs in China that were 3.2-fold higher during 2017–2018 than during 2011–2012 (*25*). *A. baumannii* was the most frequent bacterial isolate in ventilator-associated pneumonia in China, and rates were 35.7%–52.7% (*26*,*27*). Furthermore, the incidence of the drug-resistant phenotype of *A. baumannii* is high. According to CHINET reports, carbapenem-resistant *A. baumannii* strains increased from 31% in 2005 to 66.7% in 2014 (*28*), then to ≈80% in 2018 (*29*). We previously reported that carbapenem resistance rates in ICUs in China increased from 25% during 2011–2012 to 95.7% during 2017–2018 (*25*). A multicenter study of ICUs in China reported that multidrug-resistant (MDR) *A. baumannii* was detected in 40% of all cases (*30*).

We hypothesized that increased use of glucocorticoid aerosol therapy might contribute to increased *A. baumannii* incidence. Therefore, we performed a prospective cohort study of critically ill patients receiving invasive mechanical ventilation in China to determine whether use of aerosolized glucocorticoid increased the risk for *A. baumannii* isolation.

## Methods

### Study Design and Patients

During January 2018–August 2019, we conducted a prospective cohort study at 3 adult ICUs in 2 hospitals in Shandong Province, China: Qilu Hospital of Shandong University in Jinan and Qingdao Branch of Qilu Hospital in Qingdao. We enrolled patients on their first day of invasive mechanical ventilation in the ICU and obtained written informed consent for all patients. We divided the patients into 3 groups on the basis of their treatment: no aerosol inhalation therapy, glucocorticoid aerosol therapy, and aerosol inhalation without glucocorticoid. Within 48 hours of patient enrollment, we collected secretion samples from the lower respiratory tract by transtracheal aspiration for microbial culture; thereafter, we collected samples 3 times per week until we obtained an *A. baumannii–*positive culture. We followed patients for 30 days after enrollment. If the patient was hospitalized for >3 weeks, we reduced the culture frequency to once a week. We excluded patients who received invasive mechanical ventilation for <48 hours; received aerosol inhalation or glucocorticoid aerosol for <48 hours after enrollment and before *A. baumannii–*positive culture; were <18 years of age; were assumed to have *A. baumannii* infection or colonization at baseline because they were *A. baumannii*–positive before enrollment or within the first 48 hours of enrollment; or had been exposed to >1 of the following *A. baumannii* risk factors before enrollment: antimicrobial drugs for >7 days, invasive mechanical ventilation for >5 days, or vasopressor for >3 days. We also excluded patients who lacked follow-up data or had incomplete information. The Institutional Ethics Committee of Qilu Hospital of Shandong University approved our study.

### Microbiology

We performed microbial cultures according to standard procedures. In brief, we incubated respiratory samples on MacConkey agar plates at 5% CO_2_ and 35°C for 48 h. We identified *A. baumannii*, a gram-negative, nonfermentative, and oxidase-negative coccobacillus, by using the VITEK 2 compact system and GN ID card (bioMérieux, https://www.biomerieux.com). We used *Escherichia coli* (ATCC accession no. 25922) and *Pseudomonas aeruginosa* (ATCC accession no. 27853) as quality controls.

### Definitions and Data Collection

We defined no aerosol inhalation as patients who did not receive aerosolized medications during the study. We defined glucocorticoid aerosol therapy as patients who received aerosolized glucocorticoids for >48 hours after enrollment and before *A. baumannii* isolation, with or without nonglucocorticoid aerosolized medications for any duration. We defined aerosol inhalation without glucocorticoid as patients who received only aerosolized nonglucocorticoid medications, such as bronchodilators and expectorants, for >48 hours after enrollment and before *A. baumannii* isolation. We excluded all other conditions.

The primary endpoint was *A. baumannii* isolation, which we defined as *A. baumannii–*positive culture from the lower respiratory tract samples collected during the ICU stay. Negative outcomes were no *A. baumannii* isolation before death, ICU discharge, or end of follow-up period. We recorded the time-to-event, which we defined as number of days from enrollment to *A. baumannii* isolation.

We collected baseline information at ICU admission, including age, sex, history of smoking and surgeries, underlying conditions, past inhaled steroids for chronic conditions, and Charlson comorbidity index. We used the Acute Physiology and Chronic Health Evaluation II (APACHE II) score to assess illness severity. We also recorded other possible *A. baumannii* risk factors, such as use of broad-spectrum antimicrobial drugs, invasive mechanical ventilation, urethral catheter placement, vasopressor treatment, renal dialysis, and length of ICU stay. In addition, we recorded indications for glucocorticoid aerosol therapy by reviewing patients’ medical records. We reviewed patients’ clinical data to determine *A. baumannii* isolation status as infection, colonization, or undefined.

### Statistical Analyses

We expressed continuous variables as median and interquartile range (IQR) or mean and SD and categorical variables as number and percentage. We used univariate and multivariate Cox proportional hazards regression and hazard ratio (HR) and 95% CI to assess risk factors for *A. baumannii* isolation and 30-day mortality. We performed propensity score matching analysis to reduce the imbalance between the glucocorticoid aerosol therapy and nonglucocorticoid groups. We included all possible covariables (i.e., demographics, background history, underlying conditions, and disease severity) in the propensity score matching. We calculated propensity scores by using a logistic regression model. We applied a 1:1 nearest neighbor matching algorithm with a caliper of 0.02 and without replacement. We assessed balance of variables in both groups by standardized differences. We analyzed *A. baumannii* isolation in complete cases and the propensity-matched cohort. We used Kaplan-Meier curves to visually compare cumulative hazards for *A. baumannii* isolation among the 3 groups, which we evaluated by using a log-rank test. To assess the consistency of glucocorticoid therapy in terms of its effect on *A. baumannii* isolation from prespecified subgroups with different characteristics, we applied Cox proportional hazards model with Efron’s method for handling ties and used forest plots for HRs and 95% CIs. We assessed heterogeneity of efficacy of glucocorticoid therapy on *A. baumannii* isolation in subgroups by using an interaction test, expressed p values for interaction, and considered p<0.05 statistically significant. We performed all analyses by using SPSS Statistics 16.0 (IBM, https://www.ibm.com) and R version 3.0 (R Foundation for Statistical Computing, https://www.r-project.org).

## Results

### Participant Characteristics

**Figure 1:**
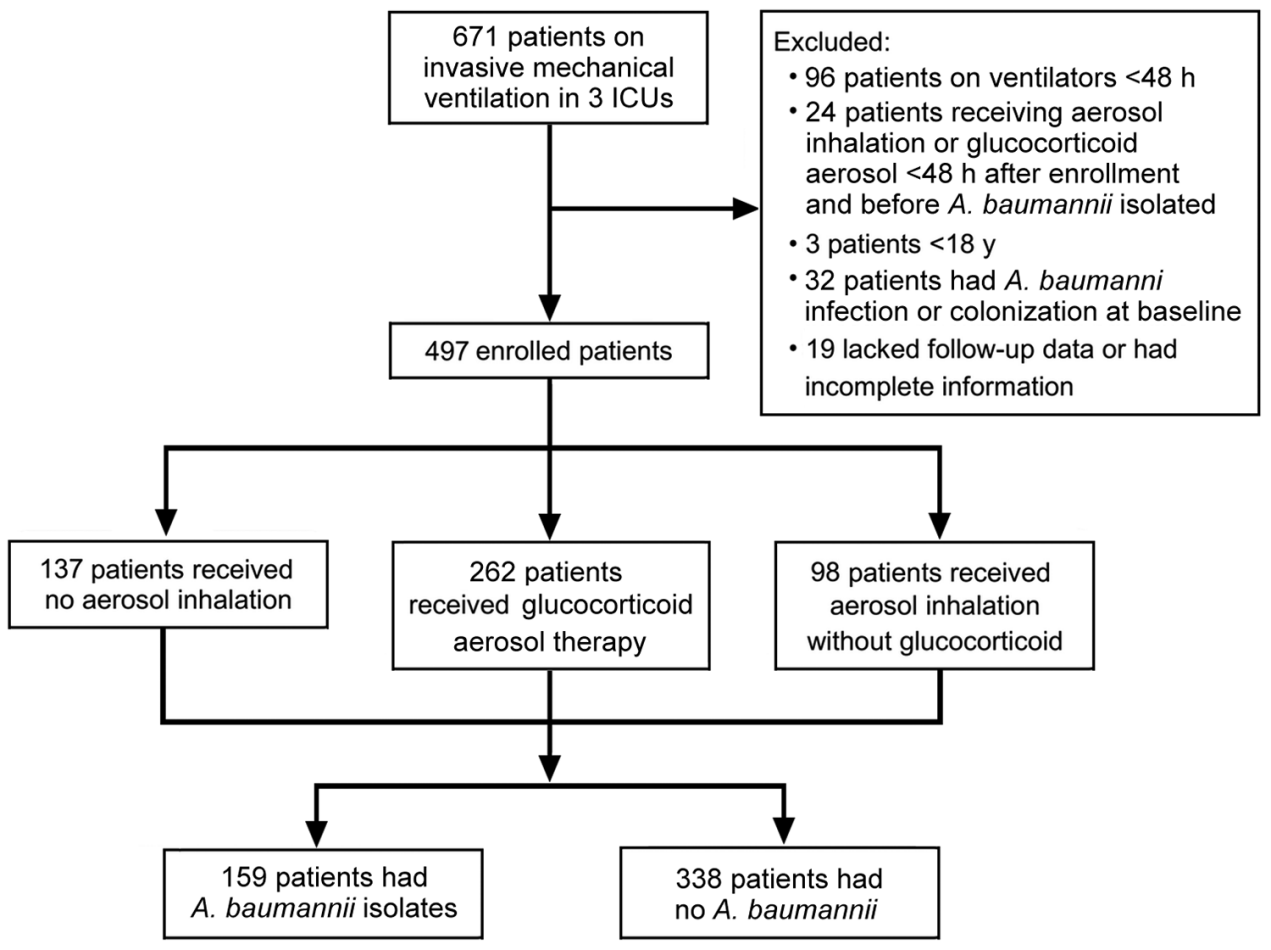
Flowchart for enrolling participants in a study of *Acinetobacter baumannii* among patients receiving glucocorticoid aerosol therapy during invasive mechanical ventilation, China. ICU, intensive care units.

We enrolled 671 patients from 3 ICUs and excluded 174 patients. The final cohort consisted of 497 patients: 137 (27.6%) received no aerosol inhalation, 262 (52.7%) received glucocorticoid aerosol, and 98 (19.7%) received aerosol inhalation without glucocorticoid (Figure 1). We isolated *A. baumannii* from 159 (32.0%) patients. The median patient age was 60.1 (IQR 49–73) years, and 67.8% were male. The median length of ICU stay was 15 (IQR 7–23) days. Besides *A. baumannii*, the 3 other bacteria commonly isolated were *Klebsiella pneumoniae* (n = 38, 7.6%), *P. aeruginosa* (n = 27, 5.4%), and *E. coli* (n = 15, 3.0%) (Appendix Table 1). Most (22.5%) study patients received glucocorticoid therapy for ARDS and for asthma or COPD (21.3%) (Appendix Table 2).

**Table 1:** Univariate analysis of risk factors for *Acinetobacter baumannii* among patients during invasive mechanical ventilation, China*

Variables	Overall, n = 497	*Acinetobacter baumannii* isolated		p value	Hazard ratio (95% CI)
		Yes, n = 159	No, n = 338		
Median age, y (IQR)	60.1 (49–73)	61 (50–74)	59.6 (49–73)	0.444	1.004 (0.994–1.013)
Sex, no. (%)					
F	160 (32.2)	44 (27.7)	116 (34.3)	Referent	
M	337 (67.8)	115 (72.3)	222 (65.7)	0.139	0.769 (0.544–1.089)
Mean Charlson comorbidity index, (SD)	4.26 (2.12)	4.40 (2.14)	4.20 (2.11)	0.293	1.038 (0.968–1.113)
Underlying conditions, no. (%)					
Cardiovascular diseases	200 (40.2)	79 (49.7)	121 (358)	0.003	1.596 (1.169–2.178)
Chronic renal insufficiency	179 (36.0)	71 (44.7)	108 (32.0)	0.011	1.504 (1.200–2.056)
COPD and asthma	176 (35.4)	71 (44.7)	105 (31.1)	0.005	1.570 (1.148–2.146)
Type 2 diabetes mellitus	116 (23.3)	46 (28.9)	70 (20.7)	0.057	1.395 (0.990–1.965)
Solid tumor	100 (20.1)	36 (22.6)	64 (18.9)	0.363	1.188 (0.820–1.723)
Hematologic malignancy	31 (6.2)	8 (5.0)	23 (6.8)	0.497	0.781 (0.384–1.591)
Past inhaled steroids use for chronic conditions	47 (9.5)	17 (10.7)	30 (8.9)	0.450	1.214 (0.734–2.007)
Current or former smoker	187 (37.6)	74 (46.5)	113 (33.4)	0.005	1.565 (1.146–2.138)
Postoperative admission	142 (28.6)	38 (23.9)	104 (30.8)	0.134	0.757 (0.526–1.090)
Treatment, no. (%)					
No aerosol inhalation	137 (27.6)	33 (20.8)	104 (30.8)	Referent	
Glucocorticoid aerosol inhalation	262 (52.7)	107 (67.3)	155 (45.9)	0.002	1.860 (1.264–2.738)
Aerosol inhalation without glucocorticoid	98 (19.7)	19 (11.9)	79 (23.4)	0.337	0.760 (0.433–1.332)
Broad-spectrum antimicrobial drugs, >7 d	417 (83.9)	157 (98.7)	260 (76.9)	<0.001	9.539 (4.595–18.795)
Invasive mechanical ventilation, >5 d	221 (44.5)	112 (70.4)	109 (32.2)	<0.001	3.452 (2.453–4.858)
Urethral catheter placement, >3 d	493 (99.2)	158 (99.4)	335 (99.1)	0.875	1.171 (0.164–8.361)
Vasopressor treatment, >3 d	75 (15.1)	42 (26.4)	33 (9.8)	<0.001	2.634 (1.850–3.750)
Renal dialysis, >3 d	84 (16.9)	34 (21.4)	50 (14.8)	0.063	1.432 (0.980–2.093)
APACHE II score, mean (SD)	18.18 (6.03)	18.98 (6.44)	17.80 (5.80)	0.053	1.026 (1.000–1.053)
Median length of ICU stay, d (IQR)	15 (7–23)	20 (10–28)	13 (6–20)	0.057	1.005 (1.000–1.010)

*APACHE II, Acute Physiology and Chronic Health Evaluation II; COPD, chronic obstructive pulmonary disease; ICU, intensive care unit; IQR, interquartile range.

**Table 2:** Multivariate analysis of risk factors for *Acinetobacter baumannii* among patients during invasive mechanical ventilation, China*

Variables	p value	Hazard ratio (95% CI)
Underlying conditions		
Cardiovascular diseases	0.054	1.394 (0.994–1.955)
Chronic renal insufficiency	0.730	0.937 (0.648–1.356)
COPD and asthma	0.132	1.299 (0.924–1.825)
Type 2 diabetes mellitus	0.325	1.197 (0.837–1.714)
Current or former smoker	0.098	1.307 (0.951–1.797)
Treatment		
No aerosol inhalation	Referent	
Glucocorticoid aerosol inhalation	0.038	1.528 (1.024–2.278)
Aerosol inhalation without glucocorticoid	0.524	0.829 (0.467–1.475)
Broad-spectrum antimicrobial drugs, >7 d	0.001	7.238 (2.758–15.788)
Invasive mechanical ventilation, >5 d	0.001	2.381 (1.664–3.405)
Vasopressor treatment, >3 d	<0.001	2.060 (1.402–3.028)
Renal dialysis, >3 d	0.841	1.046 (0.675–1.620)
APACHE II score	0.586	0.992 (0.965–1.020)

*Results are from model 2; only variables with p<0.1 in univariate analysis were included. APACHE II, Acute Physiology and Chronic Health Evaluation II; COPD, chronic obstructive pulmonary disease.

### Risk Factors for *A. baumannii* Isolation

We performed univariate Cox regression analysis of risk factors for *A. baumannii* isolation (Table 1). Compared with no aerosol inhalation, glucocorticoid aerosol therapy had a statistically significant effect on *A. baumannii* isolation (HR 1.860, 95% CI 1.264–2.738; p = 0.002). Aerosol inhalation without glucocorticoid was not a risk factor for *A. baumannii* (p>0.05). Other candidate risk factors were cardiovascular diseases, chronic renal insufficiency, COPD or asthma, current or former smoking history, use of broad-spectrum antimicrobial drugs for >7 days, invasive mechanical ventilation for >5 days, vasopressor treatment, renal dialysis for >3 days, and APACHE II score.

To assess whether glucocorticoid aerosol therapy was an independent risk factor for *A. baumannii* isolation, we established 2 models using multivariate Cox regression analysis in complete cases. Model 1 included all variables, and model 2 only included variables with p<0.1 in the univariate analysis. Glucocorticoid aerosol was an independent risk factor for *A. baumannii* isolation in both model 1 (HR 1.499, 95% CI 1.001–2.246; p = 0.049) (Appendix Table 3) and model 2 (HR 1.528, 95% CI 1.024–2.278; p = 0.038) (Table 2). Cardiovascular diseases, prolonged use of broad-spectrum antimicrobial drugs, invasive mechanical ventilation, and vasopressor treatment were other independent risk factors for *A. baumannii* (Table 2; Appendix Table 3). As a whole variable, aerosol inhalation had no effect on *A. baumannii* isolation (Appendix Table 4).

In the propensity-matched cohort, the possible glucocorticoid-related covariables were balanced in both groups (Appendix Table 5). Univariate and multivariate Cox regression analyses also indicated that glucocorticoid aerosol therapy was an independent risk factor for *A. baumannii* isolation (Table 3; Appendix Table 6). In an independent model that included indications for glucocorticoid aerosol therapy, risk factors for *A. baumannii* isolation were glucocorticoid aerosol treatments for COPD or asthma and for ARDS (Appendix Table 7).

**Table 3:** Multivariate analysis of risk factors for *Acinetobacter baumannii* among propensity-matched patient cohort during invasive mechanical ventilation, China*

Variables	p value	Hazard ratio (95% CI)
Underlying conditions		
Cardiovascular diseases	0.117	1.361 (0.926–2.001)
Chronic renal insufficiency	0.800	1.052 (0.712–1.554)
Type 2 diabetes mellitus	0.243	1.271 (0.850–1.899)
Current or former smoker	0.051	1.442 (0.998–2.083)
Treatment		
Glucocorticoid aerosol inhalation	0.032	1.489 (1.036–2.141)
Broad-spectrum antimicrobial drugs, >7 d	0.004	6.315 (2.543–13.921)
Invasive mechanical ventilation, >5 d	<0.001	2.388 (1.614–3.534)
Vasopressor treatment, >3 d	0.501	1.188 (0.719–1.963)
APACHE II score	0.363	1.014 (0.984–1.045)

*Only variables with p<0.1 in univariate analysis of the propensity-matched cohort were included. APACHE II, Acute Physiology and Chronic Health Evaluation II; COPD, chronic obstructive pulmonary disease.

**Figure 2:**
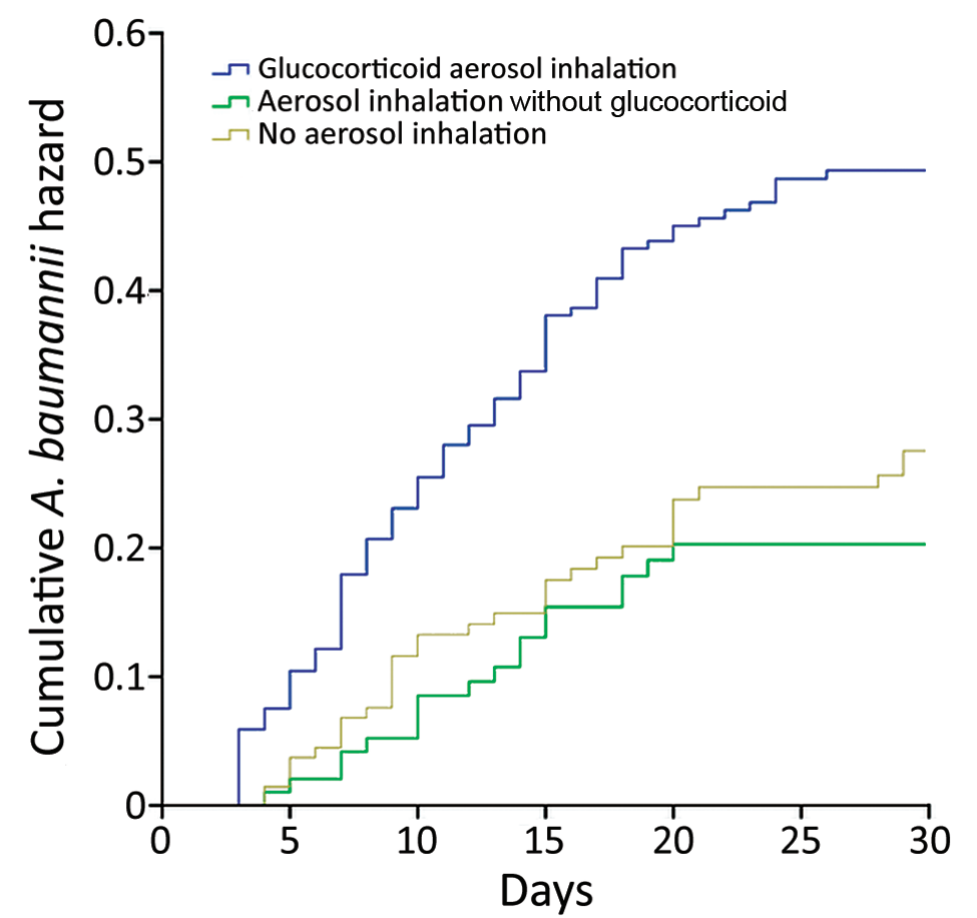
Kaplan-Meier curves of cumulative hazards of different aerosol inhalation treatments on *Acinetobacter baumannii* isolation among patients during invasive mechanical ventilation, China. We used log-rank analysis to compare hazard ratios over time among all groups (p<0.001), glucocorticoid aerosol therapy group with the no aerosol inhalation group (p<0.001), and the glucocorticoid aerosol therapy group with the aerosol inhalation without glucocorticoid group (p = 0.002).

Log-rank analysis showed that the difference among the groups was statistically significant (p<0.001). The cumulative hazard for *A. baumannii* isolation was significantly higher in the glucocorticoid aerosol group compared with the no aerosol inhalation (HR 1.871; 95% CI 1.206–2.772; p<0.001) and aerosol inhalation without glucocorticoid (HR 2.316; 95% CI 1.482–3.620; p = 0.002) groups (Figure 2).

### Effect of Glucocorticoid Aerosol Therapy on *A. baumannii* Isolation among Subgroups

**Figure 3:**
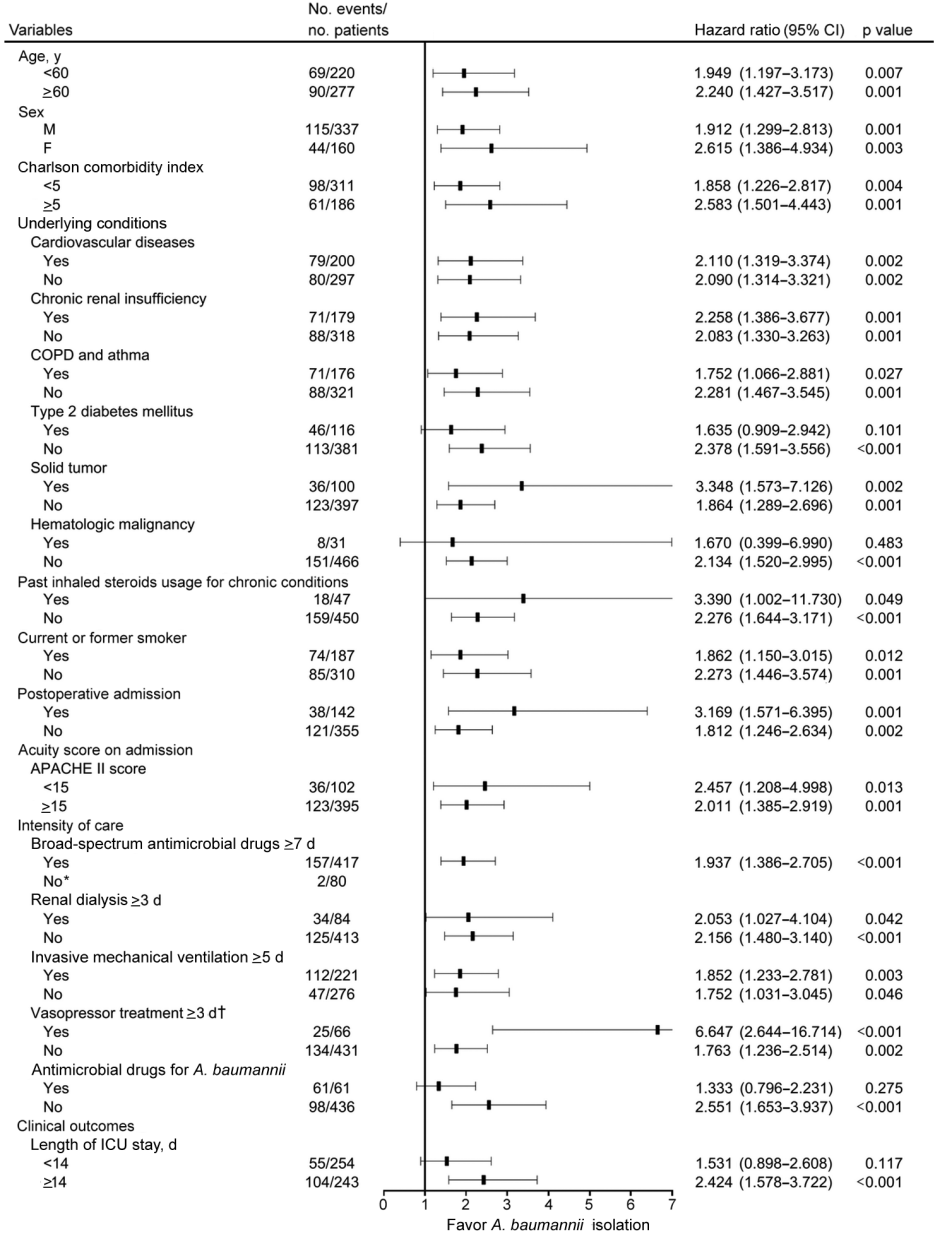
Effects of glucocorticoid aerosol therapy on *Acinetobacter baumannii* isolation during invasive mechanical ventilation among prespecified patient subgroups, China. We applied Cox proportional hazards model with Efron’s method for handling ties between groups to assess favorable eﬀects of glucocorticoid aerosol on *A. baumannii* isolation. Black horizontal marks indicate hazard ratios, and error bars indicate 95% CIs. APACHE II, Acute Physiology and Chronic Health Evaluation II; COPD, chronic obstructive pulmonary disease; ICU, intensive care unit. *Limited numbers for analysis. †For interaction, p = 0.006.

We divided patients into subgroups to evaluate the contribution of glucocorticoid aerosol to *A. baumannii* isolation from different subpopulations. We found glucocorticoid aerosol was a promoting factor for *A. baumannii* isolation from most subpopulations, except patients with type 2 diabetes mellitus, hematologic malignancy, antimicrobial drug use for *A. baumannii*, and short ICU stays (p>0.05) (Figure 3). We noted no statistically significant interactions between most prespecified subgroups defined by demographics, medical history, underlying conditions, APACHE II score, treatment measures, and length of ICU stay (interaction p>0.05). The favorable eﬀect of glucocorticoid aerosol on *A. baumannii* isolation was relatively greater in the subgroup of patients with longer vasopressor treatment (interaction p = 0.006) (Figure 3).

### Association between Glucocorticoid Aerosol Therapy and Clinical Prognosis

We performed univariate and multivariate Cox regression analyses to evaluate the possible risk factors for 30-day mortality in critically ill patients on ventilators. We found glucocorticoid aerosol was not a risk factor for 30-day mortality in those patients, but *A. baumannii* isolation was independently associated with 30-day mortality (HR 1.824, 95% CI 1.317–2.104; p = 0.045) (Appendix Tables 8, 9). A further separate analysis of *A. baumannii* isolation status showed that *A. baumannii* infection was independently associated with 30-day mortality (HR 2.759, 95% CI 1.575–4.833; p = 0.012) (Appendix Tables 8, 10).

## Discussion

In this study, we assessed the effect of the commonly used glucocorticoid aerosol therapy on the frequency of *A. baumannii–*positive cultures from lower respiratory tract samples in 3 ICUs in China. After controlling for other variables, our findings showed that glucocorticoid aerosol increased the risk for *A. baumannii* isolation from critically ill patients on invasive mechanical ventilation.

*A. baumannii* is ubiquitous in nature and is becoming more frequent in hospitals. In our study, 32% of patients acquired *A. baumannii* during the 30-day follow-up period. Over the past 2 decades, several studies have attempted to characterize and identify risk factors for *A. baumannii* colonization or infection. Invasive operations, such as endotracheal mechanical ventilation, inserted invasive devices, ICU stays, recent surgery, use of broad-spectrum antimicrobial drugs, ineffective antimicrobial therapy, and septic shock at diagnosis, are risk factors for MDR *A. baumannii* colonization or infection and for death (*5*,*31*–*33*). 

We determined that prolonged use of broad-spectrum antimicrobial drugs, invasive mechanical ventilation, and vasopressor treatment were independent risk factors for *A. baumannii* isolation from ventilated patients, which is consistent with previous studies (*5*,*31*–*33*). A previous study reported that cardiovascular organ failure was an independent risk factor associated with *A. baumannii* bloodstream infection (*34*). Of note, model 1 of our study showed that cardiovascular disease also was an independent risk factor for *A. baumannii* isolation. Another population-based study reported that patients with chronic heart failure had a markedly increased risk for hospitalization with pneumonia (*35*), indicating a possible correlation between cardiovascular diseases and pneumonia. However, the specific underlying mechanisms by which cardiovascular disease promotes *A. baumannii* isolation remain unknown.

In critically ill patients undergoing mechanical ventilation, aerosol inhalation is a common intervention for treating various pulmonary diseases. An international survey demonstrated that 99% of 611 ICUs from 70 countries reported using aerosol therapy during mechanical ventilation, including noninvasive ventilation, and the most frequently delivered drugs were bronchodilators and steroids (*36*). A web-based survey involving 447 hospitals in mainland China recorded a high proportion of aerosol therapy in both invasive (90.8%) and noninvasive (91.3%) mechanical ventilation; bronchodilators (64.8%) and topical corticosteroids (43.4%) were the most commonly used drugs (*9*). Aerosol inhalation is aimed at reversing bronchoconstriction, decreasing the work of breathing, relieving dyspnea, modifying the inflammatory response (*19*,*37*), ameliorating lung injury (*38*), and reducing the rate of exacerbation in both asthma and COPD. However, in patients with COPD and asthma, inhaled corticosteroids are associated with an increased risk for upper respiratory tract infections (*39*,*40*), pneumonia, and lower respiratory tract infections (*15*,*41*).

Ventilated patients are already vulnerable to pneumonia. Therefore, evaluating whether commonly used aerosol therapy increases the risk for nosocomial pneumonia is crucial, especially when inhaled with glucocorticoids. Because *A. baumanni–*related pneumonia is associated with severe illness and death, we chose *A. baumannii* isolation as an outcome and explored its relationship with glucocorticoid aerosol therapy. Our study showed that glucocorticoid aerosol therapy was an independent risk factor for *A. baumannii* isolation from patients on ventilators. Compared with no aerosol inhalation, glucocorticoid aerosol inhalation increased the risk for *A. baumannii* by ≈1.5 times. Although further analysis revealed that glucocorticoid aerosol was not directly associated with 30-day mortality, it still might contribute to poor clinical prognosis due to its effect on *A. baumannii* isolation. As we described, *A. baumannii*, especially MDR *A. baumannii* pneumonia, is well recognized as a risk factor for death. In this study, we also found *A. baumannii* isolation was an independent risk factor for 30-day mortality in patients receiving invasive mechanical ventilation. Because glucocorticoid aerosol heightened the likelihood of acquiring *A. baumannii*, it might exert a secondary effect, death among *A. baumannii–*infected patients. Thus, further investigation in a much larger patient population could describe a downstream mortality effect of glucocorticoid aerosol therapy. 

When we included glucocorticoid aerosol indications in multivariate analysis, we found COPD and asthma and possible ARDS were independent risk factors for *A. baumannii* isolation. Because these structural or underlying lung diseases and severe acute lung injury necessitate longer duration of mechanical ventilation, our results were compatible with previously described risk factors for *A. baumannii* infection. In contrast to glucocorticoid aerosol, we did not detect an association between aerosol inhalation without glucocorticoid and *A. baumannii*. Because both therapies generate aerosols, our previous concern that aerosols were a source of *A. baumannii* acquisition might not be reasonable.

Reasons why glucocorticoids increase the risk for *A. baumannii* isolation remain elusive. Previously considered sterile, healthy lungs harbor complex and dynamic microbiota communities (*42*). Pulmonary diseases, such as COPD (*43*), asthma (*44*), lung cancer (*45*), and ARDS (*46*), cause considerable alteration of lung microbiota. Pneumonia pathogenesis involves an abrupt and emergent disruption in the complex homeostasis of the lung microbial ecosystem (*47*). A recent study reported that inhaled corticosteroids altered the lung microbiota in both COPD patients and mouse models and impaired bacterial control in models with *Streptococcus pneumonia* infection (*14*). Antimicrobial peptides, also known as host defense peptides, are short and generally positively charged peptides that participate in the regulation of the host’s antibacterial actions and immune defense (*48*). Another study observed that cathelicidin, an antimicrobial peptide, was impaired by inhaled corticosteroids among COPD patients by increasing protease cathepsin D, thereby promoting the proliferation of *Streptococcus* (*14*). In a bacterial 16S rRNA gene sequencing and host transcriptomic analysis, another study reported that as COPD severity increased, the airway microbiome becomes associated with decreased abundance of *Prevotella* bacteria in concert with downregulation of genes promoting epithelial defense associated with inhaled corticosteroid use (*49*). Evidence also suggests that in asthma, inhaled corticosteroids can alter the relative abundance of genera in airway microbiome (*50*). Therefore, inhaled corticosteroids play a primary role in lung microbiota disruption and host-defense suppression, which could explain why glucocorticoid aerosol contributed to the increased risk for *A. baumannii* isolation from patients on ventilators.

Clinicians should individualize patient care and manage treatments on the basis of subgroup analysis results. Our results showed that, in most subpopulations, regardless of the presence or absence of the prespecified characteristics, patients were at higher risk for *A. baumannii* isolation when receiving glucocorticoid aerosol. Based on our findings, we recommend that intensive care teams more carefully consider the risk of widespread usage of glucocorticoid aerosol in patients with invasive mechanical ventilation. Because glucocorticoid aerosol therapy had a much greater favorable eﬀect on *A. baumannii* isolation in the subgroup of patients on vasopressors for >3 days, clinicians should be particularly cautious about giving glucocorticoid aerosol to patients on long vasopressor treatments. Because patients with diabetes, hematologic malignancy, and shorter ICU stays were at relatively lower risk for glucocorticoid aerosol–associated *A. baumannii* acquisition, glucocorticoid aerosol could be considered when appropriate for indications in these patients. Altogether, our study suggests ICU teams need to identify the specific patient subgroups that will truly benefit from glucocorticoid aerosol therapy rather than more generalized administration. Limiting glucocorticoid aerosol use might be considered as part of existing antimicrobial stewardship bundles. In addition, defining the duration of glucocorticoid aerosol therapy might help maximize benefit while reducing associated risk. Interventional studies exploring the effects of different glucocorticoid aerosol therapy durations on the occurrence of various types of nosocomial pneumonia are needed. 

The first limitation of our study is that, because it was an observational study, confounders that might influence the effect of glucocorticoid aerosol on *A. baumannii* isolation remain, even after adjusting by subgroup analysis, propensity score matching analysis, and multivariable Cox regression. A similar situation exists for the analysis of risk factors for death. Second, a potential time bias remains, in which patients might have been exposed to risk factors before study enrollment. However, we have excluded patients with prolonged exposure to several well-known risk factors to minimize the possible effect of time bias. Third, exclusion of certain cases might pose a potential selection bias, including survivorship bias. Future randomized controlled interventional studies are expected to confirm our findings and minimize the effects of confounders and the above biases. Fourth, the study included only 3 ICUs in China and did not focus on pathogens other than *A. baumannii*. Research involving more centers and more cases could explore whether glucocorticoid aerosol is a common risk factor in the overall nosocomial pneumonia risk.

In conclusion, we found glucocorticoid aerosol therapy was an independent risk factor for *A. baumannii* isolation from patients receiving invasive mechanical ventilation. Because of high mortality rates associated with *A. baumannii–*related nosocomial pneumonia, clinicians should carefully consider both the beneficial and harmful effects of glucocorticoid aerosol before administering this therapy.

Appendix Additional information on *Acinetobacter baumannii* among patients receiving glucocorticoid aerosol therapy during invasive mechanical ventilation, China.
