# Supplementary material for: Acinetobacter baumannii among Patients Receiving Glucocorticoid Aerosol Therapy during Invasive Mechanical Ventilation, China
Source: Emerg Infect Dis. 2022 Dec;28(12):2398–408. doi: 10.3201/eid2812.220347 (PMC9707605; doi:10.3201/eid2812.220347)
Supplement: Appendix — Additional information on Acinetobacter baumannii among patients receiving glucocorticoid aerosol therapy during invasive mechanical ventilation, China. [file 22-0347-Techapp-s1.pdf]

Article DOI: https://doi.org/10.3201/eid2812.220347

Acinetobacter baumannii among Patients Receiving Glucocorticoid Aerosol Therapy during Invasive Mechanical Ventilation, China

Appendix

Appendix Table 1. Other pathogens detected from MacConkey agar plates in a study of *Acinetobacter baumannii* among patients during invasive mechanical ventilation, China*

Bacteria	Total (%), n = 497	No. of isolates (%) Glucocorticoid aerosol therapy, n = 262	No glucocorticoid aerosol therapy, n = 235	p value
Klebsiella pneumoniae	38 (7.6)	23 (8.8)	15 (6.4)	0.316
Pseudomonas aeruginosa	27 (5.4)	16 (6.1)	11 (4.7)	0.484
Escherichia coli	15 (3.0)	9 (3.4)	6 (2.6)	0.566
Stenotrophomonas maltophilia	7 (1.4)	4 (1.5)	3 (1.3)	0.885
Burkholderia cepacia	3 (0.6)	1 (0.4)	2 (0.9)	0.925
Serratia marcescens	2 (0.4)	1 (0.4)	1 (0.4)	0.527
Others	4 (0.8)	3 (1.1)	1 (0.4)	0.694

Appendix Table 2. Indications for glucocorticoid aerosol therapy in a study of *Acinetobacter baumannii* among patients during invasive mechanical ventilation, China*

Variables	Total, n = 262	*A. baumannii* isolated, n = 107	No *A. baumannii* isolated, n = 155	p value	Hazard ratio (95% CI)
Asthma and COPD	106 (21.3)	41 (25.8)	65 (18.9)	0.087	1.400 (0.952–2.059)
ARDS	112 (22.5)	44 (27.7)	68 (20.1)	0.076	1.408 (0.965–2.052)
Other conditions†	30 (6.0)	14 (8.8)	16 (4.7)	0.112	1.592 (0.897–2.827)
Undetermined	14 (2.8)	8 (5.0)	6 (1.8)	0.115	1.801 (0.867–3.744)

*Data are expressed as no. (%). ARDS, acute respiratory distress syndrome; COPD, chronic obstructive pulmonary disease.

†Other conditions included recorded airway hyperresponsiveness, hyperinflammation or mucosal edema following diseases other than asthma, COPD, and ARDS.

Appendix Table 3. Multivariate analysis of risk factors for *Acinetobacter baumannii* isolation among patients during invasive mechanical ventilation, China*

Variables	p value	Hazard ratio (95% CI)
Age, y	0.987	1.000 (0.988–1.012)
Sex		
F	–	Referent
M	0.748	0.936 (0.624–1.403)
Charlson comorbidity index	0.115	0.920 (0.830–1.020)
Underlying conditions		
Cardiovascular diseases	0.031	1.530 (1.040–2.250)
Chronic renal insufficiency	0.703	1.080 (0.727–1.605)
COPD and asthma	0.082	1.393 (0.959–2.024)
Type 2 diabetes mellitus	0.108	1.379 (0.932–2.040)
Solid tumor	0.084	1.455 (0.950–2.229)
Hematologic malignancy	0.252	0.655 (0.317–1.352)
Past inhaled steroid usage for chronic conditions	0.654	0.868 (0.467–1.612)
Current or former smoker	0.191	1.273 (0.886–1.828)
Postoperative admission	0.581	0.899 (0.616–1.312)
Treatment		
No aerosol inhalation	–	Referent
Glucocorticoid aerosol therapy	0.049	1.499 (1.001–2.246)
Aerosol inhalation without glucocorticoid	0.571	0.846 (0.474–1.508)
Broad-spectrum antimicrobial drugs for ≥7 d	0.001	9.436 (2.803–18.661)
Invasive mechanical ventilation for ≥5 d	<0.001	2.325 (1.616–3.344)
Urethral catheter placement for ≥3 d	0.737	0.707 (0.093–5.352)
Vasopressor treatment for ≥3 d	<0.001	2.034 (1.379–3.001)
Renal dialysis for ≥3 d	0.746	1.077 (0.687–1.688)
APACHE II score	0.755	0.995 (0.967–1.025)

*Results are based on model 1 in which all variables are included. APACHE II, Acute Physiology and Chronic Health Evaluation II; COPD, chronic obstructive pulmonary disease; –, not applicable.

Appendix Table 4. Multivariate analysis of risk factors for *Acinetobacter baumannii* isolation among patients during invasive mechanical ventilation, China*

	Model 1		Model 2	
Variables	p value	Hazard ratio (95% CI)	p value	Hazard ratio (95% CI)
Age, y	0.947	1.000 (0.988–1.013)	ND	ND
Sex				
F	–	Referent	ND	ND
M	0.633	0.907 (0.608–1.354)	ND	ND
Charlson comorbidity index	0.086	0.913 (0.824–1.013)	ND	ND
Underlying conditions				
Cardiovascular diseases	0.036	1.510 (1.027–2.219)	0.060	1.386 (0.987–1.948)
Chronic renal insufficiency	0.609	1.110 (0.745–1.653)	0.782	0.949 (0.654–1.376)
COPD and asthma	0.087	1.387 (0.954–2.019)	0.121	1.309 (0.931–1.839)
Type 2 diabetes mellitus	0.109	1.379 (0.931–2.043)	0.358	1.183 (0.826–1.694)
Solid tumor	0.075	1.476 (0.962–2.266)	ND	ND
Hematologic malignancy	0.205	0.626 (0.303–1.292)	ND	ND
Past inhaled steroid usage for chronic conditions	0.824	0.932 (0.504–1.726)	ND	ND
Current or former smoker	0.215	1.255 (0.877–1.795)	0.107	1.299 (0.945–1.785)
Postoperative admission	0.462	0.868 (0.595–1.266)	ND	ND
Treatment				
No aerosol inhalation	–	Referent	–	Referent
Aerosol inhalation	0.106	1.391 (0.932–2.077)	0.089	1.413 (0.949–2.103)
Broad-spectrum antimicrobial drugs for ≥7 d	0.001	8.845 (2.903–18.339)	0.001	8.591 (2.844–17.246)
Invasive mechanical ventilation for ≥5 d	0.001	2.386 (1.659–3.431)	0.001	2.442 (1.707–3.494)
Urethral catheter placement for ≥3 d	0.671	0.645 (0.086–4.859)	ND	ND
Vasopressor treatment for ≥3 d	<0.001	2.141 (1.453–3.154)	<0.001	2.157 (1.468–3.169)
Renal dialysis for ≥3 d	0.614	1.122 (0.717–1.758)	0.726	1.081 (0.698–1.675)
APACHE II score	0.628	0.993 (0.964–1.022)	0.522	0.991 (0.964–1.019)

*We considered aerosol inhalation as a whole variable. Model 1 includes all variables; model 2 only includes variables for which p<0.1 in univariate analysis. APACHE II, Acute Physiology and Chronic Health Evaluation II; COPD, chronic obstructive pulmonary disease; ND, not done; –, not applicable.

Appendix Table 5. Univariate analysis of patients with or without glucocorticoid aerosol therapy after propensity score adjustment in a study of *Acinetobacter baumannii* isolation among patients during invasive mechanical ventilation, China*

Variables	Glucocorticoid aerosol therapy, n = 199	No glucocorticoid aerosol therapy, n = 199	p value
Median age, y (IQR)	59.9 (49–72)	60.1 (49–72)	0.899
Sex, no. (%)			
F	61 (30.7)	64 (32.2)	–
M	138 (69.3)	135 (67.8)	0.527
Mean Charlson comorbidity index (SD)	4.18 (2.04)	4.23 (2.19)	0.795
Underlying conditions, no. (%)			
Cardiovascular diseases	79 (39.7)	79 (39.7)	1.000
Chronic renal insufficiency	73 (36.7)	68 (34.2)	0.600
COPD and asthma	65 (32.7)	68 (34.2)	0.750
Type 2 diabetes mellitus	46 (23.1)	48 (24.1)	0.813
Solid tumor	37 (18.6)	39 (19.6)	0.799
Hematologic malignancy	14 (7.0)	12 (6.0)	0.685
Past inhaled steroid usage for chronic conditions	17(8.5)	15 (7.5)	0.712
Current or former smoker	75 (37.7)	73 (36.7)	0.836
Postoperative admission	59 (29.6)	58 (29.1)	0.912
Treatment, no. (%)			
Broad-spectrum antimicrobial drugs for ≥7 d	170 (85.4)	167 (83.9)	0.676
Invasive mechanical ventilation for ≥5 d	84 (42.2)	82 (41.2)	0.839
Urethral catheter placement for ≥3 d	197 (99.0)	198 (99.5)	0.562
Vasopressor treatment for ≥3 d	23 (11.6)	25 (12.6)	0.758
Renal dialysis for ≥3 d	37 (18.6)	32 (16.1)	0.508
Mean APACHE II score (SD)	18.47 (6.23)	18.05 (5.72)	0.485
Acinetobacter baumannii isolated, no (%)	70 (35.2)	51 (25.6)	0.038
Median length of ICU stay, d (IQR)	21 (9–24)	18 (5–21)	0.262

*APACHE II, Acute Physiology and Chronic Health Evaluation II; COPD, chronic obstructive pulmonary disease; ICU, intensive care unit; IQR, interquartile range; SD, standard deviation; –, not applicable.

Appendix Table 6. Univariate analysis of risk factors for *Acinetobacter baumannii* isolation among patients during invasive mechanical ventilation, China*

Variables	*A. baumannii* isolated, n = 121	No *A. baumannii* isolated, n = 277	p value	Hazard ratio (95% CI)
Median age, y (IQR)	59.2 (49–71)	60.4 (49–73)	0.460	0.996 (0.986–1.006)
Sex, no. (%)				
F	35 (28.9)	90 (32.5)	–	Referent
M	86 (71.1)	187 (67.5)	0.527	0.881 (0.595–1.305)
Mean Charlson comorbidity index (SD)	4.36 (2.06)	4.14 (2.14)	0.295	1.043 (0.964–1.129)
Underlying conditions, no. (%)				
Cardiovascular diseases	58 (47.9)	100 (36.1)	0.021	1.520 (1.064–2.172)
Chronic renal insufficiency	57 (47.1)	84 (30.3)	0.002	1.750 (1.224–2.501)
COPD and asthma	47 (38.8)	86 (31.0)	0.171	1.291 (0.896–1.861)
Type 2 diabetes mellitus	37 (30.6)	57 (20.6)	0.038	1.505 (1.022–2.215)
Solid tumor	25 (20.7)	51 (18.4)	0.563	0.878 (0.565–1.364)
Hematologic malignancy	6 (5.0)	20 (7.2)	0.450	0.729 (0.321–1.657)
Past inhaled steroid usage for chronic conditions	7 (5.8)	25 (9.0)	0.280	0.657 (0.306–1.409)
Current or former smoker	57 (47.1)	91 (32.9)	0.008	1.627 (1.138–2.325)
Postoperative admission	32 (26.4)	85 (30.7)	0.441	0.853 (0.570–1.278)
Treatment, no. (%)				
Glucocorticoid aerosol therapy	70 (57.9)	129 (46.6)	0.038	1.438 (1.003–2.061)
Broad-spectrum antimicrobial drugs for ≥7 d	120 (99.2)	217 (78.3)	0.015	7.454 (1.925–15.582)
Invasive mechanical ventilation for ≥5 d	80 (66.1)	86 (31.0)	<0.001	3.203 (1.927–5.323)
Urethral catheter placement for ≥3 d	120 (99.2)	275 (99.3)	0.472	0.485 (0.067–3.494)
Vasopressor treatment for ≥3 d	19 (15.7)	29 (10.5)	0.010	2.224 (1.215–4.069)
Renal dialysis for ≥3 d	29 (24.0)	40 (14.4)	0.112	1.559 (0.902–2.697)
Mean APACHE II score (SD)	19.62 (6.51)	17.66 (5.63)	0.002	1.056 (1.019–1.094)
Median length of ICU stay, d (IQR)	22 (10–28)	18 (6–21)	0.205	1.004 (0.998–1.011)

*We used a propensity-matched cohort for analyses. APACHE II, Acute Physiology and Chronic Health Evaluation II; COPD, chronic obstructive pulmonary disease; ICU, intensive care unit; IQR, interquartile range; SD, standard deviation; –, not applicable.

Appendix Table 7. Multivariate analysis of risk factors for *Acinetobacter baumannii* isolation among patients during invasive mechanical ventilation, China*

Variables	p value	Hazard ratio (95% CI)
Underlying conditions		
Cardiovascular diseases	0.010	1.530 (1.109–2.112)
Chronic renal insufficiency	0.852	0.967 (0.676–1.382)
Type 2 diabetes mellitus	0.654	1.084 (0.761–1.544)
Current or former smoker	0.099	1.292 (0.953–1.753)
Treatment		
No aerosol inhalation	–	Referent
Glucocorticoid aerosol for asthma and COPD	0.024	1.774 (1.077–2.920)
Glucocorticoid aerosol for ARDS	0.003	3.589 (1.955–6.589)
Glucocorticoid aerosol for other conditions or undetermined	0.060	1.705 (0.990–2.669)
Aerosol inhalation without glucocorticoid	0.170	0.690 (0.406–1.172)
Broad-spectrum antimicrobial drugs for ≥7 d	0.001	4.677 (2.036–10.743)
Invasive mechanical ventilation for ≥5 d	<0.001	2.247 (1.588–3.179)
Vasopressor treatment for ≥3 d	0.001	2.703 (1.798–4.066)
Renal dialysis for ≥3 d	0.643	1.107 (0.720–1.702)
APACHE II score	0.256	0.985 (0.959–1.011)

*Calculations include indications for glucocorticoid aerosol therapy and only include variables with p<0.1 in univariate analysis. APACHE II, Acute Physiology and Chronic Health Evaluation II; ARDS, acute respiratory distress syndrome; COPD, chronic obstructive pulmonary disease; ICU, intensive care unit; –, not applicable.

Appendix Table 8. Univariate analysis of risk factors for 30-d mortality in a study of *Acinetobacter baumannii* isolation among patients during invasive mechanical ventilation, China*

Variables	Total, n = 497	Died, n = 100	Survived, n = 397	p value	Hazard ratio (95% CI)
Median age, y (IQR)	60.1 (49–73)	59 (48–76)	61 (49–73)	0.673	0.998 (0.986–1.009)
Sex, no. (%)					
F	160 (32.2)	34 (34.0)	126 (31.7)	–	Referent
M	337 (67.8)	66 (66.0)	271 (68.3)	0.716	1.080 (0.714–1.633)
Mean Charlson comorbidity index (SD)	4.26 (2.12)	4.58 (1.96)	4.18 (215)	0.082	1.075 (0.991–1.167)
Underlying conditions, no. (%)					
Cardiovascular diseases	200 (40.2)	46 (46.0)	154 (38.8)	0.098	1.329 (0.897–1.970)
Chronic renal insufficiency	179 (36.0)	51 (51.0)	128 (32.2)	0.001	2.004 (1.354–2.967)
COPD and asthma	176 (35.4)	39 (39.0)	137 (34.5)	0.331	1.220 (0.817–1.824)
Type 2 diabetes mellitus	116 (23.3)	25 (25.0)	91 (22.9)	0.521	1.160 (0.738–1.824)
Solid tumor	100 (20.1)	23 (23.0)	73 (18.4)	0.327	1.262 (0.792–2.011)
Hematologic malignancy	31 (6.1)	11 (10.4)	20 (5.0)	0.022	2.075 (1.109–3.884)
Past inhaled steroid usage for chronic conditions	47 (9.5)	13 (13.0)	34 (8.6)	0.153	1.530 (0.854–2.740)
Current or former smoker	187 (37.6)	37 (37.0)	150 (37.8)	0.839	0.959 (0.639–1.439)
Postoperative admission	142 (28.6)	21 (21.0)	121 (30.5)	0.100	0.649 (0.401–1.050)
Treatment, no. (%)					
No aerosol inhalation	137 (27.6)	33 (33.0)	104 (26.2)	–	Referent
Glucocorticoid aerosol therapy	262 (52.7)	49 (49.0)	213 (53.7)	0.087	0.753 (0.484–1.170)
Aerosol inhalation without glucocorticoid	98 (19.7)	18 (18.0)	80 (20.2)	0.389	0.777 (0.438–1.380)
Broad-spectrum antimicrobial drugs for ≥7 d	417 (83.9)	85 (85.0)	332 (83.6)	0.928	1.026 (0.592–1.778)
Invasive mechanical ventilation for ≥5 d	220 (44.3)	62 (62.0)	158 (39.8)	0.001	2.079 (1.388–3.114)
Urethral catheter placement for ≥3 d	493 (99.2)	99 (99.0)	394 (99.2)	0.701	0.950 (0.680–4.873)
Vasopressor treatment for ≥3 d	75 (15.1)	52 (52.0)	23 (5.8)	<0.001	8.988 (6.048–13.357)
Renal dialysis for ≥3 d	84 (16.9)	22 (22.0)	62 (15.6)	0.163	1.401 (0.873–2.248)
Mean APACHE II score (SD)	18.18 (6.03)	20.07 (7.10)	17.70 (5.64)	0.001	1.056 (1.025–1.089)
A. baumannii isolation, no. (%)	159 (32.0)	47 (47.0)	112 (28.2)	0.001	1.977 (1.335–2.929)
A. baumannii isolation status, no. (%)					
No *A. baumannii* isolation	338 (68.0)	53 (53.0)	285 (71.8)	–	Referent
A. baumannii colonization	96 (19.3)	21 (21.0)	75 (18.9)	0.251	1.228 (0.720–2.097)
A. baumannii infection	58 (11.7)	25 (25.0)	33 (8.3)	0.001	3.346 (2.115–5.293)
A. baumannii colonization or infection not defined	5 (1.0)	1 (1.0)	4 (1.0)	0.755	1.371 (0.190–9.911)
Antimicrobial drugs treatment for *A. baumannii*	65 (13.1)	18 (18.0)	47 (11.8)	0.102	1.565 (0.943–3.297)

*APACHE II, Acute Physiology and Chronic Health Evaluation II; ARDS, acute respiratory distress syndrome; COPD, chronic obstructive pulmonary disease; –, not applicable.

Appendix Table 9. Multivariate analysis of risk factors for 30-d mortality in a study of *Acinetobacter baumannii* isolation among patients during invasive mechanical ventilation, China*

Variables	p value	Hazard ratio (95% CI)
Charlson comorbidity index	0.380	0.953 (0.855–1.062)
Underlying conditions		
Cardiovascular diseases	0.491	1.164 (0.756–1.791)
Chronic renal insufficiency	0.420	1.208 (0.763–1.914)
Hematologic malignancy	0.154	1.616 (0.836–3.127)
Treatment		
No aerosol inhalation	–	Referent
Glucocorticoid aerosol therapy	0.102	0.750 (0.497–1.065)
Aerosol inhalation without glucocorticoid	0.388	1.308 (0.711–2.408)
Invasive mechanical ventilation for $\geq$5 d	0.386	1.226 (0.774–1.942)
Vasopressor treatment for $\geq$3 d	<0.001	8.366 (5.314–13.170)
APACHE II score	0.114	1.026 (0.994–1.060)
A. baumannii isolation	0.045	1.824 (1.317–2.104)

*Calculations include *A. baumanni* isolation as a whole variable and only variables with p<0.1 in univariate analysis were included. APACHE II, Acute Physiology and Chronic Health Evaluation II; –, not applicable.

Appendix Table 10. Multivariate analysis of risk factors for 30-d mortality in a study of *Acinetobacter baumannii* isolation among patients during invasive mechanical ventilation, China*

Variables	p value	Hazard ratio (95% CI)
Charlson comorbidity index	0.490	0.962 (0.863–1.073)
Underlying conditions		
Cardiovascular diseases	0.941	1.016 (0.657–1.572)
Chronic renal insufficiency	0.491	1.176 (0.741–1.869)
Hematologic malignancy	0.132	1.657 (0.859–3.195)
Treatment		
No aerosol inhalation	–	Referent
Glucocorticoid aerosol therapy	0.106	0.747 (0.493–1.066)
Aerosol inhalation without glucocorticoid	0.404	1.296 (0.705–2.382)
Invasive mechanical ventilation for ≥5 d	0.729	1.088 (0.675–1.756)
Vasopressor treatment for ≥3 d	0.001	8.989 (5.720–14.126)
APACHE II score	0.148	1.023 (0.992–1.055)
A. baumannii isolation status		
No *A. baumannii* isolation	–	Referent
A. baumannii colonization	0.720	0.894 (0.484–1.650)
A. baumannii infection	0.012	2.759 (1.575–4.833)
A. baumannii colonization or infection not defined	0.488	0.494 (0.067–3.634)

* Calculations include four *A. baumanni* isolation status and only variables with p<0.1 in univariate analysis were included. APACHE II, Acute Physiology and Chronic Health Evaluation II; –, not applicable.
